# Development, Implementation, and Effectiveness of a Self-sustaining, Web-Based LGBTQ+ National Platform: A Framework for Centralizing Local Health Care Resources and Culturally Competent Providers

**DOI:** 10.2196/17913

**Published:** 2021-09-22

**Authors:** Dustin Z Nowaskie

**Affiliations:** 1 Department of Psychiatry Indiana University School of Medicine Indianapolis, IN United States

**Keywords:** cultural competency, disparities, e-health, healthcare, internet, LGBTQ+, online platform, providers, resources, eHealth, health care

## Abstract

**Background:**

The lesbian, gay, bisexual, transgender, queer, and other sexual and gender minority (LGBTQ+) population has long faced substantial marginalization, discrimination, and health care disparities compared to the cisgender, heterosexual population. As the etiology of such disparities is multifaceted, finding concrete solutions for LGBTQ+ health care equity is challenging. However, the internet may offer the space to initiate an effective model.

**Objective:**

In an effort to make LGBTQ+ public resources and culturally competent providers transparent, modernize medical education, and promote cultural competency, OutCare Health—a nonprofit 501(c)(3) multidisciplinary, multicenter web-based platform—was created.

**Methods:**

The organization employs a cyclic, multidimensional framework to conduct needs assessments, identify resources and providers, promote these efforts on the website, and educate the next generation of providers. LGBTQ+ public health services are identified via the internet, email, and word of mouth and added to the Public Resource Database; culturally competent providers are recruited to the OutList directory via listservs, medical institutions, local organizations, and word of mouth; and mentors are invited to the Mentorship Program by emailing OutList providers. These efforts are replicated across nearly 30 states in the United States.

**Results:**

The organization has identified over 500 public health organizations across all states, recognized more than 2000 OutList providers across all states and 50 specialties, distributed hundreds of thousands of educational materials, received over 10,000 monthly website visits (with 83% unique viewership), and formed nearly 30 state-specific teams. The total number of OutList providers and monthly website views has doubled every 12-18 months. The majority of OutList providers are trained in primary, first point-of-care specialties such as family medicine, infectious disease, internal medicine, mental health, obstetrics and gynecology, and pediatrics.

**Conclusions:**

A web-based LGBTQ+ platform is a feasible, effective model to identify public health resources, culturally competent providers, and mentors as well as provide cultural competency educational materials and education across the country. Such a platform also has the opportunity to reach self-perpetuating sustainability. The cyclic, multidisciplinary, multidimensional, multicenter framework presented here appears to be pivotal in achieving such growth and stability. Other organizations and medical institutions should heavily consider using this framework to reach their own communities with high-quality, culturally competent care for the LGBTQ+ population.

## Introduction

### Background

The lesbian, gay, bisexual, transgender, queer, and other sexual and gender minority (LGBTQ+) population has long confronted substantial stigma and discrimination within public and health care settings [1,2]. Numerous studies have revealed that LGBTQ+ people are more likely to endure health care disparities and have poorer physical and mental health outcomes than their cisgender, heterosexual counterparts [3-5].

The etiology of such LGBTQ+ disparities is multifaceted. Upwards of 40% of LGBTQ+ patients experience health care discrimination such as stigmatizing attitudes, refusal of needed medications, and verbal and physical violence [1]. These experiences then lead to postponements and avoidances of routine and urgent care due to anticipation and fear of reliving such stigma [1,2,6]. Likewise, the LGBTQ+ population faces more financial barriers to health care than the cisgender, heterosexual population [7,8].

Health care professionals are stationed to both understand these health care complexities as well as intervene when appropriate to alleviate and prevent poor health outcomes. However, many students and providers have been shown to display both explicit and implicit biases [9,10] and discriminatory attitudes [1,2,11], infrequently collect sexual orientation and gender identity information [12-14], and demonstrate shortcomings in education and cultural competency [13-16]. Notably, the quality of LGBTQ+ medical education and the amount of time spent on it has improved only marginally since the 1990s [17], as medical students in 2009-2010 received a median of only five hours of “fair” LGBTQ+ education across their four years of training [18].

Finding concrete solutions for LGBTQ+ health care disparities is challenging. Identifying methods to eliminate discriminatory health care encounters may lead to better health outcomes for LGBTQ+ patients. Institutional endeavors such as increasing formal LGBTQ+ education and curricular reform have shown promising benefits for provider knowledge and attitudinal awareness [19]; however, these educational initiatives, at this time, are locally concentrated and are not standardized or universal. When considering a national solution for LGBTQ+ health equity, the internet may offer the space to initiate an effective model. Past research has shown that LGBTQ+ people use social media and online sources at a higher rate than cisgender, heterosexual individuals [20]. Additionally, the internet serves as an important avenue for sexual expression and health information gathering for LGBTQ+ people [21-23]. Therefore, a national online platform that serves the LGBTQ+ population has the potential to alleviate health care disparities by improving LGBTQ+ health care equality and equity. For example, an online platform affords an excellent opportunity to increase the following: (1) visibility and accessibility of culturally competent care by identifying vetted public resources, providers, and mentors; (2) awareness and appreciation of health care disparities and gaps in provider knowledge by conducting evidence-based academic research; and (3) cultural competency by training the current and next generation of providers.

### Objectives

In an effort to make LGBTQ+ public resources and culturally competent providers transparent, modernize medical education, and promote cultural competency, OutCare Health [24]—a nonprofit 501(c)(3), multidisciplinary, multicenter online platform—was created. Ever since its foundation and initiation, the organization’s overarching goals include health care equality and equity for all people regardless of sexual orientation, gender identity, race, ethnicity, socioeconomic status, and other social classifications; access to health care information; collaboration among health care providers and organizations; and a broad community of support for the LGBTQ+ population. Particular objectives include the following: (1) increasing visibility of public health resources and culturally competent providers for the general public; (2) providing medical education and consultation for curricular reform; and (3) creating and distributing educational materials, providing cultural competency trainings, and hosting seminars and conferences for health care providers.

## Methods

OutCare Health was founded in May 2015. The organization employs a cyclic, multidimensional framework to conduct needs assessments, identify resources and providers, promote these efforts on the website, and educate the next generation of providers. For instance, needs assessments, such as evaluating LGBTQ+ patients’ satisfaction with medical care [25] and characterizing providers’ [13,26,27] and students’ [28,29] attitudes, practices, and knowledge, through self-reported surveys are conducted at local and national levels. At the same time, LGBTQ+ public health services are identified via the internet, email, and word of mouth and added to the Public Resource Database; culturally competent providers are recruited to the OutList directory via listservs, medical institutions, local organizations, and word of mouth; and mentors (who provide consultation on school, career, research, and/or other academic pursuits for students, staff, and/or faculty) are invited to the Mentorship Program by emailing OutList providers. Cultural competency trainings are then delivered in person locally and online nationally to fill gaps in LGBTQ+ clinical preparedness, attitudinal awareness, and basic knowledge. Trainings incorporate both clinical (eg, terminology and disparities) and nonclinical (eg, stigma, microaggressions, and how to create welcoming environments) components. Additionally, state-specific team members champion the groundwork for change by creating and distributing educational materials, such as brochures, references, and referrals, to students, faculty, staff, providers, public organizations, and health care systems as well as by promoting the Public Resource Database, OutList, and Mentorship Program locally. Members also meet with curriculum committees to implement educational reform. Although this framework is operated at local levels by state-specific teams, the online organization serves as a centralized source for all of these social and public health efforts.

## Results

**Figure 1:**
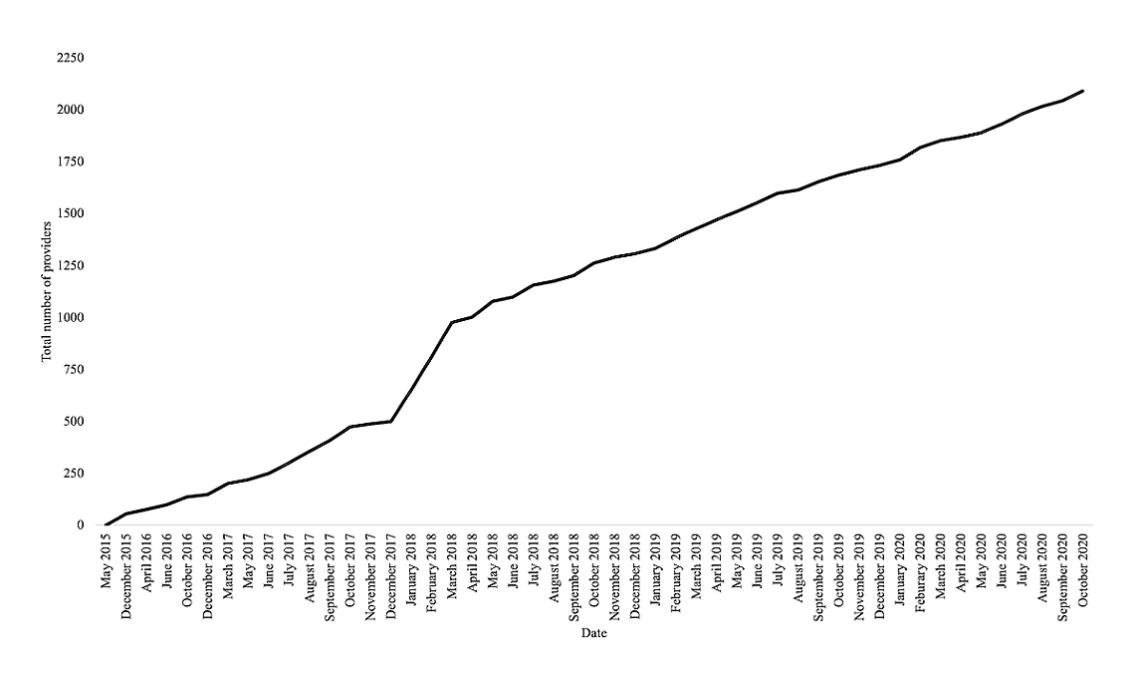
OutCare Health OutList growth. Growth of the total number of culturally competent providers in OutCare Health’s OutList provider directory. The number of culturally competent providers was calculated via online submission count and Google Analytics. Data between OutCare Health’s initiation (May 2015) and April 2017 were intermittently collected.

**Figure 2:**
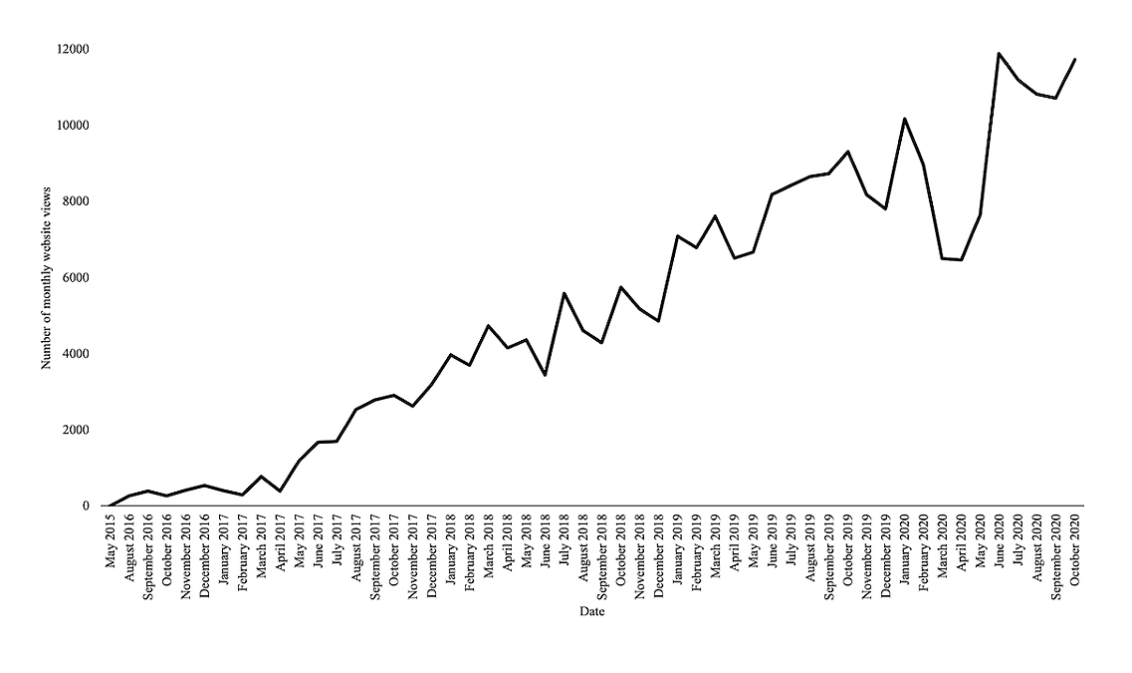
OutCare Health website's viewership growth. Growth of monthly website views on OutCare Health. The number of monthly views was calculated via Google Analytics (83% are unique viewers). Data were not collected between OutCare Health’s initiation (May 2015) and July 2016.

The organization’s impact to date includes the following: identifying over 500 public health organizations across all states on the Public Resource Database, recognizing more than 2000 culturally competent providers across all states and 50 specialties on the OutList, distributing hundreds of thousands of educational materials, receiving over 10,000 monthly website visits (with 83% unique viewership), and forming nearly 30 state-specific teams. Both the total number of OutList providers and monthly website views have doubled every 12-18 months (Figure 1, Figure 2). Within the OutList, there are more primary, first point-of-care specialties represented than other health care specialties; these include family medicine (n=370), infectious disease (n=86), internal medicine (n=218), mental health and counseling (n=731), obstetrics and gynecology (n=161), pediatrics (n=137), psychiatry (n=78), and social work (n=118).

## Discussion

### Principal Findings

Health care disparities are substantially prevalent among the LGBTQ+ population. Many studies have demonstrated that health care providers can express negative attitudes and erroneous beliefs about LGBTQ+ people. Incorrect assumptions can lead to inadequate care if providers do not have the awareness and knowledge of how LGBTQ+ cultural factors impact health. Thus, LGBTQ+ health care equity requires providers who will impart and advocate for the care of and respect for the LGBTQ+ population in a culturally competent manner while providing safe spaces.

In an effort to make LGBTQ+ resources transparent, modernize medical education, and promote cultural competency, the nonprofit organization OutCare Health implements an online cyclic, multidisciplinary, multidimensional, multicenter framework to foster such change. Longitudinal projects, such as identifying public health resources, culturally competent providers, and mentors, has allowed the organization to promote awareness and up-to-date information and education so that the current and future health care workforce can deliver better LGBTQ+ care. The growth of the Public Resource Database, OutList, Mentorship Program, and website viewership highlights the necessity of this valuable information for public and health care communities. Of note, while there are some continued efforts to increase use of these databases via direct communication, the organization has become a self-sustaining online platform. For example, of the monthly viewers, a high percentage are new visitors to the website. Likewise, the majority of newly enlisted OutList providers practice in states that have not been directly marketed to as of yet. Given the disproportionate primary care and mental health disparities that LGBTQ+ people face, the growth of the OutList parallels this need in specialty-specific ways (ie, the majority of OutList providers are trained within family medicine, internal medicine, mental health, obstetrics and gynecology, and pediatrics). Sustainability is also achieved through providers’ ability to update and maintain their own OutList profiles. To complement this sustainability, the organization’s state-specific teams identify resources and providers within their respective states and create self-sustaining public and health care presences as well. Consequently, OutCare Health and its resources are reaching new members and spreading across communities organically. However, providing these LGBTQ+ services to particular areas and populations, such as rural communities and people without access to the internet, has proven challenging. Future efforts include collaborations with large national health care organizations as well as local LGBTQ+ public groups to improve community outreach, dissemination of this information, and access to care.

### Conclusions

Health care equity for the LGBTQ+ population is both a community and institutional endeavor. An online LGBTQ+ platform is a feasible, effective model to identify public health resources, culturally competent providers, and mentors as well as provide culturally competent educational materials and education across the country. Such a platform also has the opportunity to reach self-perpetuating sustainability. The cyclic, multidisciplinary, multidimensional, multicenter framework presented here appears to be pivotal in achieving such growth and stability. Other local organizations and medical institutions should heavily consider recognizing LGBTQ+ health care and associated disparities as a multifaceted health concern. By implementing the framework discussed here, these groups would likely be effective in reaching their own and surrounding communities with high-quality, culturally competent care for the LGBTQ+ population.

## Abbreviations

LGBTQ+: lesbian, gay, bisexual, transgender, queer, and other sexual and gender minorities
